# The New Year

**Published:** 1893-01

**Authors:** 


### HALL'S JOURNAL OF HEALTH

TRUTH DEMANDS NO SACRIFICE; ERROR CAN MAKE NONE.

Vol. 40. JANUARY, 1893. No. 1.

### THE NEW YEAR.

Once more "I stand aside to let the phantoms of the past go by me." Such is substantially the opening of one of a series of retrospects, which the inimitable Dickens wove into the story of David Copperfield.

"The phantoms of the past." How they come to the front, responsive to the bidding of our will, and fill the mind's eye with life like pictures of by-gone days. For the moment, the downward stream has turned back on itself, the same fields of activity as in other days lie before us ; the same difficulties are there to be surmounted and the same prize to be won or lost in the encounter. It is a backward view, not of a *dead* past, but of one filled with the bustle and stir of life—of old time scenes and images in the full sway of worldly endeavor, wherein circumstances repeat themselves with the exactness of a mirror. Bailey, in his Festus, somewhere says :

> " The past ! there is no past,
> And the future is a fiction of a fiction ;
> The present moment is eternity."

In a sense, this is true ; but in a very technical sense, for out of the dead past comes all that is most valuable to the ever-living present. In a word, the past lives in the present. Man alone is able to transmit to generations yet unborn, whatever of value his intellect has achieved for himself and for others, in the inexhaustible realms of literature, of science and of art, the noble trinity that should advance hand in hand along the untrodden ways of life. Thus it is that the master minds of antiquity commune with us to-day, with the familiarity of a near acquaintance. Even a twelve-month retrospect brings vividly to view many a struggling soul who has fallen by the way in the march of the "innumerable host" along the great highway whose ending is only a pause and a beginning, veiled from the observation of men.

"There is no death; what seems so is transition." Out of the past comes this assuring message as from the lips of an angel. Throughout all the changes of time and circumstance nothing is lost. Even matter is as imperishable as the faculties that mold it into untold forms for the uses of man. The hardest substance in nature may be reduced to an invisible gas and made to form new relations in its contact with other substances. A thousand changes await it, but annihilation is impossible.

Who can believe that that mysterious quality of animal life we are wont to call instinct, so near akin to reason, and oftentimes undistinguishable from it, dies with the body and counts for nothing in the universe of organized being? At the best, how little we know of nature's subtle laws! How little we know of ourselves! But we are wandering from our text. It was our intention to speak of the dawning of the New Year, and somewhat of the changes which the old year has wrought in our midst. Not so much of men and their belongings as of events which send the current of thought into new and unused channels. The ages through which the world has passed have acquired distinctive appellations significant of its progress from lower to higher planes in the various fields of industry. Hence came the age of stone, of iron, of steam, etc., and now, more than all, the electric age. The wonderful strides which have been made in mastering this hitherto untamable element and commanding its obedience, are visible upon every hand, and we have come to know that the ruling force which pervades all forms of being, as well animate as inanimate, is electrical. Man himself, in his physical construction, is an electrical machine. But over all is the will that sets the complex machinery in motion, and defies analysis. The present year, with its assemblage of all that is best in the accomplishment of the nations, and of peoples of every clime in Christendom, to a vast centre of civilization, in itself a wonder of growth unmatched in the world's history, is a testimony of the life and energy of the New World, which thus commemorates the landing of Columbus upon one of its outpost isles four hundred years ago, when the woodsman's ax first awoke the echoes of the vast primeval forest.

In the measurement of time as applied to states and countries, four hundred years is a comparatively narrow span. In contemplating it, we perceive at its beginning, the untamed savage, in his rustic wigwam, subsisting upon such of the wild game as his cunning and crude weapons are able to overcome, and at its termination, all the grand achievements which the highest civilization has gained for the comfort and amelioration of mankind.

May the God of nations preserve and prosper us in all good works, in the future as in the past.

